# Evaluation of vasovagal tonus index and electrocardiographic parameters in horses using a new modified base apex lead method

**DOI:** 10.14202/vetworld.2024.1385-1390

**Published:** 2024-06-28

**Authors:** Theerapong Pontaema, Pongphol Pongthaisong, Wootichai Kenchaiwong, Chayanon Chompoosan, Wichaporn Lerdweeraphon

**Affiliations:** 1 Applied Animal Physiology Research Unit, Faculty of Veterinary Science, Mahasarakham University, Mahasarakham, 44000, Thailand; 2 Small Ruminant Research Unit, Faculty of Veterinary Science, Mahasarakham University, Mahasarakham, 44000, Thailand; 3 Network Center for Animal Breeding and Omics Research, Khon Kaen University, Khon Kaen, 40002, Thailand

**Keywords:** electrocardiogram, evaluation, horses, vasovagal tonus index

## Abstract

**Background and Aim:**

Vasovagal tonus index (VVTI) serves as a straightforward assessment tool for autonomic function during both physiological and pathological conditions, including pregnancy, in horses. Obtaining VVTI through a modified base apex lead system could be a practical and comfortable solution. In this study, we assessed VVTI in horses with respect to training status and electrocardiographic measurements utilizing a novel modified base apex lead technique.

**Materials and Methods:**

A total of 12 Thai native crossbred horses and 12 Arabian horses, all free of cardiac abnormalities, were enrolled in the study. Animals underwent electrocardiogram (ECG) and VVTI using both the base-apex lead method and its modified version. 25 mm/s and 10 mm/mV ECG recordings provided standard bipolar limb leads. The amplitudes and durations of P waves, QRS complexes, PR interval, QT interval, and T duration were assessed. The T wave’s shape was examined. Each recording’s R-R interval was utilized to assess heart rate. Twenty consecutive beats were used to compute the variability of heart rate (VVTI).

**Results:**

The P wave amplitude was the only significant difference (p < 0.05) between the base apex lead method and the modified base apex lead method, with no variations in heart rate, P duration, PR interval, T duration, and QRS duration and amplitude. Both methods showed mainly biphasic T wave patterns. The VVTI values of all horses did not differ significantly between the base apex and modified base apex methods. There was no significant difference in VVTI between Thai crossbred horses and Arabian horses in either method.

**Conclusion:**

This study provided the first evidence that VVTI can be evaluated using the modified base apex lead system and may be useful for cardiovascular function monitoring in horses.

## Introduction

Due to the limitations of routine clinical electrocardiogram (ECG) recording, cardiac abnormalities can be challenging to diagnose [1]. Cardiac abnormalities can be difficult to investigate due to insufficient routine clinical diagnosis by ECG recording [1]. Assessing heart rate variability (HRV) in a clinical context aids the evaluation of autonomic nervous functions. It is valuable for diagnosing cardiac arrhythmias, tracking recovery, determining overtraining status, and evaluating sport horse performance [2–4]. Telemetric ECG and ambulatory Holter systems enable rapid and noninvasive HRV assessments. Telemetric electrocardiography (Holter monitoring), with ECG configuration and HRV records, is used for monitoring arrhythmias during exercise [2, 5–7]. The equipment is limited to stable rest or low-intensity exercises such as endurance for accurate evaluation in horses due to constraints like following the horse’s path or staying within a radio signal’s range [8]. The equipment is most suitable for monitoring sustained arrhythmias such as atrial fibrillation and ventricular premature beats, which need observation within 24 h [1, 9–11]. Telemetric ECG is not affordable in Thailand. A 3 or 4 electrode resting digital ECG system has non-invasive and cost-effective abilities, along with adequate sensitivity for accurate heart rate rhythm identification, as described by Van Loon [12]. It can also evaluate HRV in short-term recording or vasovagal tonus index (VVTI) (time domain analysis of HRV) for the diagnosis of stress [13], pregnancy [14], and cardiac disease [15]. The relationship between training status and VVTI in horses is undefined.

In horses, an ECG is typically performed using the base-apex lead system, which places electrodes on the skin surface based on Einthoven’s design. To obtain the largest complex on the electrode and facilitate interpretation, place the electrodes in line with the main depolarization direction, which is dorsal for ventricular activation and cranial for atrial activation [12]. This is due to the widespread Purkinje network extending throughout the ventricular myocardium, resulting in multiple points of depolarization across the myocardium, which is different from that observed in humans and small animals [12]. Optimal electrode positions for good quality ECG recordings have been extensively researched, decreasing movement artifacts and facilitating effortless interpretation [16–19]. The QRS complex amplitude and duration are unrelated to ventricular size. A four-electrode system with a modified base-apex recording could accurately diagnose heart rate and rhythm at rest. The electrodes should be placed along the main direction of depolarization, resulting in the largest complex, and will facilitate interpretation because the cardiac vector of the ventricular activation is dorsal, whereas the atrial activation is cranial with respect to the body surface [12]. The QRS complex and heart rate/VVTI measurement should be positioned vertically for optimal clinical observation and data acquisition.

Positioning electrodes vertically on the right side of a horse, with the positive at the 5^th^ intercostal space and the negative at the right jugular furrow, could potentially improve signal quality and reduce movement artifacts. This new modified base apex lead system is suitable for obtaining VVTI from racing horses, providing routine monitoring of cardiovascular function and performance.

This study aimed to assess electrocardiographic parameters and VVTI between untrained (Thai native crossbred) and trained (Arabian) horses using a new modified base apex lead method.

## Materials and Methods

### Ethical approval

All procedures performed on animals in this study were approved by the Institutional Animal Ethics Committee, Mahasarakham University, Thailand (Approval number: IACUC-MSU-52/2023).

### Study period and location

The study was conducted from May to July 2023 at the Husbandry Section of the 2^nd^ Livestock and Agriculture Division, Veterinary and Remount Department, Tha Phra Subdistrict, Mueang District, Khon Kaen Province, Thailand, Faro Farm, Mueang District, Roi Et Province, and SK Horse Stables, Mueang District, Sakon Nakhon Province, Thailand.

### Animals

A total of 24 healthy female horses, including Thai native crossbred horses (n = 12) and Arabian horses (n = 12) aged 6.0 ± 2.1 years with a body weight of 419.8 ± 51.7 kg, were used in this study. Animals without any cardiac problems, such as heart murmurs, cardiac arrhythmias, or structural heart abnormalities, were included in the study. The criteria for cardiac problems were based on the results of auscultation and resting ECG recordings and echocardiography.

### ECG examination

**Figure-1:**
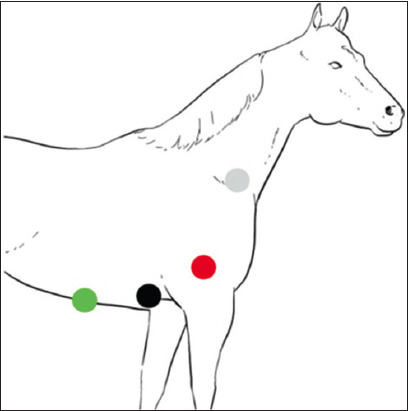
Electrode placement of the modified base apex lead method.

ECG recordings were performed on animals using a 3-channel electrocardiograph (Edan Instruments, Inc., VE-300, China) at a paper speed of 25 mm/s and calibration of 10 mm equal to 1 mV. Before recording, the animals were restrained in a standing position without any chemical restraint for acclimation within 5 min. ECG recordings for all horses were made using the base-apex lead method and immediately followed by the modified method. Four electrodes were placed on unshaved skin with alligator clips for all standard bipolar limb leads (leads I, II, and III) and unipolar augmented limb leads (lead aVR, aVL, and aVF) in 1 min. The positions of the four electrodes in the two methods are shown in Table-1 and Figure-1. Alcohol was applied to improve electrical contact. The ECG was recorded for approximately 1 min for each lead system. The recording from lead I was used to evaluate heart rate (HR) and ECG parameter measurements, including P wave duration and amplitude, PR interval, QRS duration and amplitude, and QT interval. The T wave morphology was observed with positive and negative deflection and biphasic patterns. Heart rate was evaluated using the R-R interval. All analyses of ECG recordings were performed by the same researcher.

**Table-1:** Electrode placement.

Electrode	Base apex lead method	Modified base apex lead method
LA (black)	At 5^th^ intercostal space, just behind the point of the elbow of the left forelimb	At 5^th^ intercostal space, just behind the point of the elbow of the right forelimb
RA (white)	At the right jugular furrow or in front of right scapula spine	Same position in Base apex lead method
LF (red)	On the loose skin at the left stifle in the region of the patella	On the skin at the triceps brachii of the right forelimb
RF (green or ground electrode)	On the loose skin at the right stifle in the region of the patella	On the skin at the xiphoid process

LA=Left arm, RA=Right arm, LF=Left foot, RF=Right foot

### Calculation of the HRV

All horses were evaluated for VVTI in this study. VVTI is a time-domain indicator of HRV analysis. VVTI measurement used short-term recordings and may be appropriate for resting horses. Good quality ECG traces and continuous running of sinus rhythm at the first 20 consecutive R-R intervals of 1 min in each ECG recording were selected for HRV measurement. HRV (VVTI) was obtained by calculating the variance standard deviation of the R-R interval2 for this interval in milliseconds and the natural logarithm of the variance of the 20 measured R-R intervals, as described by the equation: VVTI = NL (VAR [R-R1 − R-R20]), where NL is the natural logarithm and VAR is the variance.

### Statistical analysis

All data were analyzed using an independent t-test with SAS software University Edition (SAS, Inc., Cary, NC, USA), and p < 0.05 was considered statistically significant. The variation in ECG parameters between the two methods was examined to obtain the percentage of coefficient of variation (%CV). Descriptive statistics of the VVTI used a Kruskal–Wallis test. p < 0.05 was considered statistically significant.

## Results

**Figure-2:**
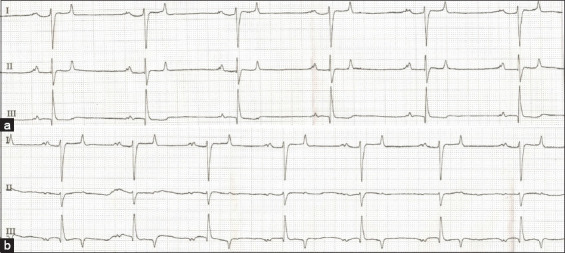
Example of electrocardiogram recording in lead I, II, and III for (a) the base apex lead method (b) and the modified base apex lead method from the same horse (paper speed = 25 mm/s, sensitivity = 10 mm/mV).

The ECG parameters are presented with their respective mean, standard deviation, and 95% confidence interval (Table-2). ECG parameters were determined using the base apex lead and the modified base apex lead system, as depicted in Figures-2a and b. The P wave amplitude differed significantly between the two methods (p < 0.05). The P wave’s amplitude was lower in the modified base apex method compared to the base apex method. The other ECG parameters, including HR, P-wave duration, PR interval, QRS duration and amplitude, QT interval, and T-wave duration, showed no significant difference between the two methods. The T wave displayed a predominantly biphasic morphology in both the base apex and modified base apex methods. In Table-3, there was no significant difference in the coefficient of variation of the ECG parameters between the two methods. The descriptive statistics of VVTI were equivalent between the base apex and modified base apex methods (Table-4). The VVTI in Thai crossbred and Arabian horses were comparable in both methods (Table-5).

**Table-2:** Comparison of the electrocardiographic parameters (mean ± SD) between the base apex and the modified base apex method.

ECG parameters	Base apex lead method		Modified base apex lead method		p-value
	
	Mean ± SD	95% CI (lower–upper)	Mean ± SD	95% CI (lower–upper)	
HR (beats/min)	37.44 ± 7.57	37.44–40.64	36.03 ± 7.42	36.03–39.16	0.4978
P wave duration (s)	0.13 ± 0.03	0.13–0.14	0.12 ± 0.04	0.12–0.14	0.5653
P wave amplitude (mV)	0.18 ± 0.07	0.18–0.20	0.13 ± 0.05	0.13–0.15	0.0064
PR interval (s)	0.26 ± 0.06	0.26–0.29	0.27 ± 0.06	0.27–0.29	0.9625
QRS duration (s)	0.12 ± 0.01	0.12–0.13	0.12 ± 0.02	0.12–0.13	0.8465
ORS amplitude (mV)	1.43 ± 0.33	1.43–1.57	1.45 ± 0.33	1.45–1.59	0.7918
QT interval (s)	0.47 ± 0.04	0.47–0.49	0.46 ± 0.04	0.46–0.48	0.3555
T wave duration (s)	0.12 ± 0.04	0.12–0.14	0.12 ± 0.03	0.12–0.13	0.477

SD=Standard deviation, ECG=Electrocardiogram, HR=Heart rate, CI=Confidence interval

**Table-3:** The %CV of ECG parameters between two methods.

ECG parameters	%CV		p-value of equality of variances

	Base apex lead method	Modified base apex lead method	
HR (beats/min)	20.22	20.60	0.92
P wave duration (s)	23.37	32.04	0.25
P wave amplitude (mV)	39.43	38.36	0.15
PR interval (s)	23.97	21.89	0.71
QRS duration (s)	9.27	13.78	0.07
ORS amplitude (mV)	22.86	22.39	0.99
QT interval (s)	9.23	9.46	0.99
T wave duration (s)	32.87	26.62	0.25

ECG=Electrocardiogram, HR=Heart rate, %CV=Percentage coefficient of variation

**Table-4:** Descriptive statistics of VVTI in each method.

Statistical measures	Base apex lead method (n = 24)	Modified base apex lead method (n = 24)	Kruskal-Wallis test (p-value)
Basic statistical measures			
Mean ± SD	9.18 ± 1.25	9.2070 ± 0.96	
Median	8.83	9.18	0.91
Standard error of mean	0.25	0.19	
Skewness	0.14	0.45	
Coefficient variation	13.62	10.46	
Variability			
Variance	1.56	0.93	
Range	5.46	4.16	
Interquartile Range	1.55	1.14	

VVTI=Vasovagal tonus index, SD=Standard deviation

**Table-5:** Descriptive statistics of VVTI in each method by breeds.

Statistics	Base apex lead method		Kruskal-Wallis test (p-value)	Modified base apex lead method		Kruskal-Wallis test (p-value)
	
	Thai cross breed (n = 12)	Arabian (n = 12)		Thai cross breed (n = 12)	Arabian (n = 12)	
Minimum	7.8	6.4		7.4	7.7	
25% Percentile	8.3	8.2		8.4	8.5	
Median	9.1	9.0	0.88	9.1	9.3	0.68
75% Percentile	9.9	10.4		9.6	10.0	
Maximum	11.8	11.3		11.6	10.8	
Coefficient of variation	11.39%	15.04%		11.4	8.90	

VVTI=Vasovagal tonus index

## Discussion

Both lead methods yielded similar resting HRs for horses. The study found that the modified base apex lead HR and rhythm monitoring method was equivalent to the standard method in sport horses. The electrodes were positioned more vertically to ensure clear QRS amplitude and observe the main ventricular depolarization direction. The P wave amplitude was less in the modified base apex lead method than the base apex lead method, but the P wave duration remained unchanged. The atrial activation might have proceeded in a more cranial-caudal direction instead of the main ventricular activation’s ventral-dorsal direction [12]. Atrial activity may not be optimally recorded using these electrode positions. The other ECG parameters of the modified base apex lead method, identical to previous reports [14, 16, 17], were all normal. Despite the trend of rising QRS duration variation in the modified base apex lead method, the duration proved insignificant for determining ventricular size and, thus, unnecessary for diagnosing equine cardiac hypertrophy. Instead of the standard base apex lead method, the modified base apex lead method can be used for heart rate and rhythm observation in resting ECG recording of horses.

VVTI, derived from a 20-beat time series analysis of HRV, is a simple method for evaluating sympathetic and parasympathetic control balance. In resting horses, VVTI is an appropriate method for assessing cardiac function [8, 14]. The modified base apex lead method and base apex lead method can calculate VVTI values of 9.18 and 8.83, respectively. Placing electrodes vertically on the right side according to the modified base apex lead method can assess VVTI in resting horses. This technique could simplify electrode attachment and indicate when they detach from the skin.

High-intensity training and overtraining in sport horses alter their cardiovascular function through modifications in autonomic nervous regulation [3]. HRV can be utilized to assess cardiovascular responses to various training techniques in horses. Time-domain HRV analysis methods such as VVTI have not been compared between untrained and trained horses yet. Despite the use of two different analysis methods, VVTI remained unaltered for both Thai cross-breed and Arabian horses (VVTI values: 9.1 and 9.0 for base apex lead method, and 9.1 and 9.3 for modified base apex lead method). The data suggested that VVTI remained unchanged regardless of the horses’ training status. Although VVTI uses only short-term recordings, these data are an indicator of vagal control of heart rate and may, therefore, be appropriate in the resting horse [8]. HRV analysis in the frequency domain should be performed during exercise because it is a sensitive method for assessing exercise response to cardiovascular function [20]. Thus, telemetric ambulatory ECG with portable recording and storage of ECG data is suitable for time or frequency domain analysis of HRV in long-term recordings in horses [21, 22].

## Conclusion

The new modified base-apex lead method, as shown in this study, is a dependable approach for diagnosing heart rate and rhythm in horses through VVTI analysis. Identifying the P wave configuration accurately is a limitation of this method.

## Authors’ Contributions

WK: Designed the study and analyzed the data. CC, PP, TP, and WL: Recorded and analyzed the data. WL: Coordinated the study and wrote and revised the manuscript. All authors have read, reviewed, and approved the final manuscript.
